# Effect of Prestorage Treatments and Zero‐Energy Cooling Chamber Storage on the Physicochemical Quality and Shelf Life of Orange‐Fleshed Sweet Potato Tubers

**DOI:** 10.1155/ijfo/6704324

**Published:** 2026-11-10

**Authors:** Derartu Girma Deressa, Getachew Neme Tolesa, Solomon Abera Habtegebriel, Fekireselassia Mekuria Mesfine, Dessalegn Legese Negewo, Alemayehu Hirko Muleta, Abdeta Tadesse Adugna

**Affiliations:** ^1^ Department of Chemical Engineering Jimma Institute of Technology Jimma University Jimma Ethiopia ju.edu.et; ^2^ Department of Food Technology School of Applied Sciences Faculty of Sciences The Papua New Guinea University of Technology Lae Morobe Province Papua New Guinea unitech.ac.pg; ^3^ Department of Food Technology and Process Engineering Haramaya Institute of Technology Haramaya University Dire Dawa Ethiopia haramaya.edu.et; ^4^ Department of Hydraulic and Water Resources Engineering Haramaya Institute of Technology Haramaya University Dire Dawa Ethiopia haramaya.edu.et

**Keywords:** calcium chloride, cooling chamber, hot water, storage condition, tuber crop

## Abstract

Sweet potato (*Ipomoea batatas*) is a key tuber crop, second in size and seventh in global food production. It faces significant postharvest losses, with quality declining by 45%–69% due to improper handling, including respiration, moisture loss, sprouting, and disease. This study examined how prestorage treatments and storage in a zero‐energy cooling chamber affect the storage and nutritional quality of OFSP tubers. The experimental design featured a three‐factor full factorial model, including prestorage treatments (CaCl_2_ dip, hot water, and a control), storage conditions in a zero‐energy cooling chamber, and storage times (Days 8, 15, 22, and 29). CaCl_2_‐treated OFSP tubers had the lowest weight loss (9.17%) and highest marketability (63.93%) after 29 days in a cooling chamber. On Day 29 in a zero‐energy cooling chamber, CaCl_2_‐treated sweet potato samples maintained the highest levels of crude protein (5.85%), crude fiber (2.36%), ash (3.24%), and fat (0.92%), with intermediate moisture content (72.3%) and carbohydrates (80.41%). They had the highest levels of beta‐carotene at 53.27 mg per 100 g and vitamin C at 7.15 mg per 100 g. Treating orange‐fleshed sweet potato tubers with CaCl_2_ and using ZECC storage can extend their storage life for farmers and processors.

## 1. Introduction

Sweet potato (*Ipomoea batatas* (L.) Lam.) is a perennial herbaceous crop characterized by a cluster of runner stems adorned with edible leaves and buds. There are four widely available local varieties: purple skin, white flesh, yellow skin, orange flesh, and purple skin with purple flesh [1]. It ranks as the world′s second‐largest tuber crop and the seventh‐largest food crop in terms of production [1]. The roots of sweet potato are nutritious and can have an essence of any color (white, cream, yellow, orange, or purple) with various textures [2]. Sweet potatoes provide an array of nutrients, including carbohydrates, fiber, carotenes, thiamine, riboflavin, niacin, potassium, zinc, calcium, iron, vitamins A and C, and high‐quality protein [3].

Because of their high adaptability to various farming conditions, shorter growing periods, high yields per hectare, low input requirements, and reduced risk of pest infestation, sweet potatoes have played significant roles in enhancing household and national food security, health, and the overall livelihoods of low‐income families in developing countries [3]. Additional important characteristics of sweet potatoes include their ability to withstand harsh environmental conditions such as drought, low soil fertility, high rainfall, and minimal maintenance needs. They also possess a rapid production cycle, high nutritional value, and favorable sensory qualities regarding flesh colors, taste, and texture [4].

They are excellent sources of minerals, vitamins, and antioxidants. They also contain nondigestible dietary fiber [5]. In addition to their antioxidant effects, phenolic compounds and carotenoids contribute to the distinctive flesh and skin colors of sweet potatoes (deep yellow, red to orange, purple, and light) [6]. Because of the rich array of nutritional components in orange‐fleshed sweet potato (OFSPs) that possess anticancer and cardiovascular disease (CVD)–preventive properties, scientists have underscored the significance of this food for human health [7]. Current scientific studies reveal that the phenolic acid constituents in OFSPs have advantageous health‐promoting properties, including antioxidative effects and free radical scavenging capabilities [5]. The levels of vitamin A in OFSPs available in SSA are sufficient for children under five. Additionally, sweet potatoes offer considerable energy (76 kcal/100 g), unlike many other vegetables [8].

In the postharvest chain, which extends from harvest to consumption, postharvest loss (PHL) refers to the measurable qualitative and quantitative loss of products at any stage [9]. Foods with higher moisture content and easily hydrolyzed ingredients are more susceptible to PHLs, which may manifest as weight loss, quality deterioration, value reduction, nutrient depletion, or a combination of these [10]. The hefty, uncured sweet potato roots are perishable, limiting the profitable distance over which they can be transported. After harvest, OFSP roots are vulnerable to various PHLs [6]. One of the most significant factors impacting the quality and value of the crop is improper postharvest handling practices [5]. During storage, various causal factors exacerbate PHLs by altering the tuber′s physical and chemical properties through respiration, moisture loss, sprouting, and disease transmission [11].

The zero‐energy cooling chamber (ZECC) utilizes evaporative cooling techniques to create a cool environment [12]. Constructing an evaporative cooling chamber is technologically simple, straightforward, and cost‐effective, as it can be built with readily available materials [6]. Water and air serve as the working fluids in the environmentally friendly air conditioning system known as evaporative cooling, which relies on induced processes of heat and mass transfer [13]. A ZECC can increase humidity while lowering the temperature by 10°C–15°C. These conditions are ideal for storing produce [14]. The ZECC method lowers the temperature and raises the relative humidity of the storage environment compared with ambient conditions, which suppresses enzymatic degradation and respiratory activity (softening) for fresh agricultural produce, especially fruits and vegetables [15]. This study introduces an innovative and sustainable postharvest preservation strategy that combines calcium salt or hot water prestorage treatments with ZECC storage. This integrated approach explores their synergistic effects on maintaining physicochemical quality, preserving *β*‐carotene and vitamin C, minimizing physiological deterioration, and extending the shelf life of OFSP tubers. The findings offer mechanistic insights and present a practical, low‐cost postharvest technology to reduce losses and enhance nutritional security in resource‐limited regions.

## 2. Materials and Methods

### 2.1. Experimental Materials

A 30‐kg sample of the Alamura variety of OFSPs, a new variety released by Haramaya University in 2023, was sourced from farmers in Babile, East Hararghe Zone, Oromia Regional State, Ethiopia. Roots were gathered and taken to Haramaya University′s Food Science and Postharvest Technology Laboratory, whereas calcium chloride was sourced from Merry Chemical Dealers in Addis Ababa.

### 2.2. Experimental Design

The experiment utilized a three‐factor full factorial (3 × 3) design with three replications, resulting in a total of 27 treatment combinations. A total of 63 OFSPs received a single treatment, whereas 189 OFSPs were involved in this experiment. The three factors were as follows: a ZECC, prestorage treatments (10% calcium chloride [16], hot water at 45°C for 20 minutes [17], and distilled water as a control), and storage durations (8, 15, 22, and 29 days) [9]. Three OFSPs were randomly selected from the treatment group to create sample solutions for biochemical and physical analysis of the corresponding parameters.

### 2.3. Sample Preparation

Farmers provided the OFSP roots, from which uniformly sized and shaped samples were chosen. These selected roots were then washed with tap water to remove impurities and drained on the same day.

### 2.4. Prestorage Treatments

Fresh OFSP samples were washed and divided into three portions; the first portion was then treated by immersion in a 10% (w/v) CaCl_2_ solution for 1 hour. A solution was prepared by mixing 100 g of CaCl_2_ with 1 L of water. Subsequent portions were treated differently: One was immersed in hot water (45°C for 20 minutes using a blancher), and another was washed with distilled water.

### 2.5. Storage Condition

Treated and control tubers were transported to Dire Dawa after treatment and stored in a ZECC with a sawdust cooling pad, as shown in Figure 1 below. This ZECC maintained an average relative humidity of 85.3% and a temperature of 23.3°C under ambient conditions, where the environment has a range of relative humidity (50%–75%) and the temperature (27°C–33°C). The chamber successfully drops the daily maximum temperature by 6°C–10.2°C and raises the relative humidity to a steady 85%–88%. The control tubers were stored separately in ambient conditions at the same location, which had an average relative humidity of 54.6% and temperature of 29.4°C. Figure 1 shows the design and structure of the ZECC and the actual one (all dimensions are in mm) [18].

**Figure 1:**
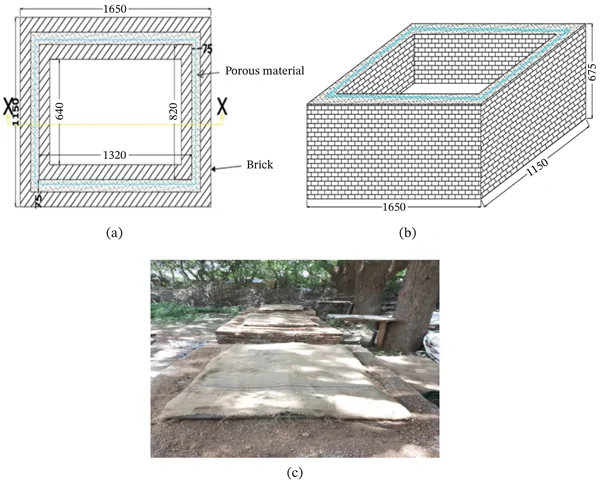
(a) The top view design of ZECC storage (dimensions are in mm). (b) The L × W × H design dimensions of ZECC storage (dimensions are in mm), ZECC′s design, and structure. (c) Actual ZECC storage [19].

### 2.6. Physiochemical Analysis

#### 2.6.1. Percentage of Weight Loss

The weight loss of the OFSP sample was determined using the Khaliq et al. [20] specified method. On the first day of the experiment, the sample in each replication was marked and weighed on a digital balance. The same sample was routinely weighed at various stages throughout the storage duration. Weight loss was determined using the equation below.

(1) Physiological weight loss=initial weight−final weightinitial weight×100%.

#### 2.6.2. Marketability

The randomly chosen samples were examined for visible surface deterioration. Based on the theory that a root becomes unmarketable when more than 10% of the surface exhibits apparent decay, the proportion of sweet potatoes deemed unmarketable due to decay was calculated for each pretreatment. Roots in storage with significant decay (more than 50% of the surface) were removed [10].

(2) Marketability=number of unmarketable sampletotal number of sample×100%.

#### 2.6.3. Decay or Rotting

All tubers from each treatment replicate were examined based on Hossain et al. [21] individually for symptoms of deterioration, including soft rot, tissue breakdown, fungal growth, discoloration, water‐soaked lesions, unpleasant odor, or any visible signs of microbial infection. Tubers exhibiting one or more of these symptoms were classified as decayed and excluded from subsequent quality assessments. The number of decayed tubers was recorded and expressed as decay incidence (%) using the formula:

(3) Decay percentage=number of decayed tuberstotal number of tubers ×100%.

#### 2.6.4. Vitamin C

The vitamin C (ascorbic acid) content of stored OFSP roots was determined using the 2,6‐dichlorophenolindophenol (DCPIP) titrimetric method. The analysis was conducted following Mekonen et al. [22]. Representative tubers from each treatment were washed, peeled, and cut into small pieces. Approximately, 10 g of fresh tissue was accurately weighed and homogenized with 50 mL of chilled 3% metaphosphoric acid (or a mixture of 3% metaphosphoric acid and 8% acetic acid) to stabilize ascorbic acid and prevent oxidative degradation during extraction. The homogenate was filtered through filter paper, and the filtrate was collected in a volumetric flask. An aliquot (10 mL) of the extract was then titrated immediately against standardized DCPIP dye until a light pink. Ascorbic acid was expressed in milligrams per 100 grams of dry weight (mg/100 g DW) in the data presented.

#### 2.6.5. Beta‐Carotene

Using AACC (2000) technique 14‐50, which is based on solvent extraction of pigments and measuring color absorbance with a UV‐visible spectrophotometer at 435.8 nm, the quantity of *β*‐carotene in fresh OFSP samples was determined. OFSP samples were dried, powdered, and tempered to 14% moisture before being sifted with a 106‐mesh sieve. Subsequently, 8‐g samples were placed in 125‐mL glass stoppered flasks, and 40 mL of reagent was added. The mixture was shaken, allowed to stand for 18 hours, shaken again, and then filtered through 0.45‐*μ*m paper into test tubes [22].

The spectrophotometer was calibrated at 435.8 nm using a reagent. Subsequently, the absorbance of washed sample extracts was measured at the same wavelength. The *β*‐carotene content was calculated (mg/g) using Johnson et al. [23] and the following equation:

(4) β−carotene mg100g=A×V×10,0002592×W,

where A is the specific absorption coefficient of *β*‐carotene in the extraction solvent (commonly 2592 in petroleum ether), V is the volume, and W is the band width (435.8 nm).

### 2.7. Proximate Composition Determination

#### 2.7.1. Moisture

Moisture content was determined by calculating the percentage difference between the original and final weights of the sample, according to Chilungo et al. [24]. Tubers were cleaned, cubed, and mixed for uniformity. About 2 g of the sample was placed in a preweighed crucible and dried in an oven at 103^°^C ± 2^°^C for 16 hours. After cooling in a desiccator, the final weight was recorded to determine moisture loss. The decrease in weight reflects the percentage of moisture content, as calculated using the equation provided below.

(5) Moisture content=W1+W2−W3+W1W2×100%,

where W_1_ is the weight of the crucible, W_2_ is the weight of the sample prior to drying, and W_3_ is the weight of the sample postdrying.

#### 2.7.2. Crude Fat Content

The Soxhlet extraction method was used as per AOAC (2005) 930.09. A 2‐g sample of sweet potato tuber flour was weighed and placed in a thimble, sealed with cotton wool. After drying, the thimble was put inside the Soxhlet extractor. Each extraction beaker was prepared by adding 200 mL of petroleum ether solvent, then dried and weighed. Beakers for extraction were dried and weighed with boiling chips. Each beaker had 200 mL of petroleum ether. Cold water flowed through the condenser to cool the system. The beaker was placed in the Soxhlet and extracted for 8 hours in the “rinsing” position and 15 minutes in the boiling position. After the solvent evaporated, the beakers were emptied and dried at 100°C for 30 minutes. The fat‐filled beaker was cooled in a desiccator before being reweighed using the equation.

(6) Ether extract=W3−W1W2×100%,

where W_3_ is the weight of beaker + weight of fat, W_2_ is the weight of sample, and W_1_ is the weight of beaker.

#### 2.7.3. Crude Protein

The AOAC (2005)′s micro‐Kjeldahl method was used to measure the protein content in the samples. Digestion: A 100‐mL micro‐Kjeldahl digestion flask was filled with 1 g of each flour. About 1 g of copper sulfate and 10 g of sodium sulfate were added, mixed well, and the flask was placed at an angle on the digesting rack. The sample was heated in a flame chamber until frothing stopped, then it was digested. When the color turned bluish‐green, the temperature was increased, and the mixture was boiled for about an hour. After digestion, the sample was cooled.

Distillation: The digested sample was mixed with distilled water and placed in a 100‐mL volumetric flask using a wash bottle. Then, 10 mL of the digest was pipetted into a micro‐Kjeldahl distillation flask, and 60 mL of a 60% sodium hydroxide (NaOH) solution was added. The flask was quickly attached to the splash head of the distillation device. Two drops of methyl red indicator and 4% boric acid were added to a 100‐mL receiving conical flask, with the delivery tube′s adapter extending below the boric acid solution′s surface. The mixture was heated to release ammonia into the receiving flask containing boric acid and an indicator until a yellowish‐green distillate was obtained. Titration: The distillate was titrated with 0.1 N hydrochloric acid (HCl) until it turned pink. The percentage (%) protein was calculated in the following equation:

(7) Protein content=T×0.00146.25×sample weight gdb×100%,

where 6.25 is the protein conversion factor, 0.0014 is the correction factor of the acid, and T represents the titer value of the sample, and db is dry base of weight of sample.

#### 2.7.4. Crude Fiber

The crude fiber content of sweet potato samples was determined according to the AOAC (2000) method. A 2‐g sample was boiled gently for 30 minutes with 200 mL of 1.25% sulfuric acid, maintaining the liquid volume with hot distilled water. Then, 20 mL of 20% KOH was added, followed by another 30 minutes of gentle boiling with occasional stirring. The digested sample was subsequently prepared for filtration using a sintered glass crucible containing a 10‐mm layer of moistened sand. The digested sample was vacuum‐filtered through a sintered glass crucible, with the beaker and residue repeatedly washed using hot distilled water. The residue was further washed sequentially with 1% sulfuric acid and 1% KOH, followed by hot distilled water and a final rinse with anhydrous acetone to remove remaining impurities. The crucible containing the residue was dried at 130°C for 2 hours, cooled in a desiccator for 30 minutes, and weighed (W_2_). It was then ashed in a muffle furnace at 550°C for 30 minutes, cooled in a desiccator, and reweighed (W_3_). The crude fiber was determined by the following equation:

(8) Crude fiber=W2−W3W1×100%,

where W_1_ represents the weight of the sample, W_2_ denotes the weight of the crucible with the sample following oven drying, and W_3_ indicates the weight of the crucible with the sample after ashing.

#### 2.7.5. Ash Content

The AOAC (2005) approved technique method Number 923.03 was used to calculate the ash content. The crucible was cleaned, dried, and cooled in a desiccator before being weighed (W_1_). Approximately 2 g of flour was added to the empty porcelain crucible, which had previously been set ablaze over a hot plate in the fume cupboard to char organic matter. The crucible was then placed in a muffle furnace and maintained at 550°C for 6 hours before being transferred to a desiccator, cooled, and weighed again immediately (W_3_).

(9) Ash content=W3−W1W2×100%,

where W_1_ denotes the weight of the crucible, W_2_ represents the weight of the sample, and W_3_ indicates the weight of the crucible plus ash.

#### 2.7.6. Utilizable Carbohydrate Content

The carbohydrate content (CHO) was determined by difference, which means 100% minus other proximate chemical compositions, using the following equation:

CHO %=100−crude protein %+fat %+ash content %+moisture content %+fiber content %.

#### 2.7.7. Data Analysis

The experimental data were analyzed using analysis of variance (ANOVA) for a three‐factor full factorial design with the SAS statistical package (Version 9.4). All samples were analyzed in replicates, and the results were expressed as mean ± standard deviation. The least significant difference (LSD) test was employed to assess the difference between means, with significance set at a probability level of (*p* < 0.05).

## 3. Results and Discussion

### 3.1. ZECC Temperature and Relative Humidity

The experimental environment in Dire Dawa, Ethiopia, featured temperatures ranging from 24.5°C to 32°C, relative humidity between 23% and 54.6%, and an air velocity of 16.3 km/h. High temperatures in an area lead to increased water evaporation and moisture retention in the air. This process converts sensible heat to latent heat, cooling the chamber and significantly raising the surrounding relative humidity [15]. Continuously watering the ZECC is crucial to keeping it cool and enhancing the ZECC′s efficiency.

### 3.2. Physiological Weight Loss (PWL)

PWL is primarily associated with respiration and moisture evaporation through the skin. The process of respiration resulted in the evaporation of H_2_O and CO_2_ generation [25]. The results indicated that weight loss increased gradually across all treatments during storage, as shown in Table 1. In samples stored under ZECC conditions, the weight loss values were 15.87% for hot water, 9.17% for CaCl_2_‐treated tubers, and 18.02% for untreated tubers, revealing significant (*p* < 0.05) differences among prestorage treatments. The low PWL in ZECC was attributed to high RH and low temperature.

**Table 1:** Interaction effect of prestorage treatments and zero‐energy cooling chamber storage on physiological weight loss (%) of OFSP tuber.

Prestorage treatment	Storage condition	Storage days			29
		8	15	22	
Control		6.90 ± 0.19^b^	10.00 ± 0.33^b^	13.59 ± 1.49^c^	18.02 ± 1.08^a^
Hot water	ZECC	5.25 ± 0.45^c^	8.47 ± 0.09^c^	12.85 ± 0.57^c^	15.87 ± 0.62^b^
CaCl_2_		4.41 ± 0.17^d^	6.48 ± 0.17^d^	7.94 ± 0.55^e^	9.17 ± 1.05^c^
LSD		0.43	1.79	1.61	1.88
CV		3.98	10.47	6.71	6.56

*Note:* Means within the same column with different letters are significantly different (*p* < 0.05).

Compared with ambient storage conditions, ZECC storage with CaCl_2_ treatment is a more effective method for retaining weight, as shown in Table 2. Both calcium chloride and hot water treatments reduced weight loss in OFSP tubers compared with control samples, with calcium chloride being the most effective across both storage methods [26]. Shin et al. [27] suggest that calcium regulates the breakdown of mitochondrial, endoplasmic reticulum, and cytoplasmic membranes, which helps to limit respiration rates and minimize spoilage. Hot water treatment was hypothesized to inactivate surface proteins and tissues in sweet potatoes, thereby reducing water evaporation and protecting against pathogens. Calcium in vegetable cell walls enhances shelf life and quality by serving as a binding agent, which delays ripening and senescence while reducing respiration rates and physiological disorders. This is why vegetables treated with calcium chloride had a longer shelf life [26]. The finding aligns with those of Sunmola and Bukoye [28] and El Wafa et al. [26], who found that CaCl_2_ prestorage treatments and hot water treatments reduced the PWL of sweet potatoes during storage life compared with the control. Calcium ions and pectin linkages in cell walls prevent water loss, leading to rigid cell walls and cytoplasm shrinkage, without affecting the fruit or vegetable′s total water content [29].

**Table 2:** ANOVA of physiological weight loss.

Source	DF	SS	Mean square	*F*	*Pr* > *F*
Pretreatments	2	167.86	83.93	308.22	< 0.0001
Storage_day	4	1392.68	348.17	1278.62	< 0.0001
Storage_method	1	13.28	13.28	48.75	< 0.0001
Storage D*pretre*storage M	6	0.42	2.51	9.25	< 0.0001
Error	54	4.70	0.27		
Corrected total	80	1703.28			

*Note:* The asterisks denote the abbreviations for "D" (day) and "pretre" (pretreatment).

### 3.3. Decay or Rotting

Under ZECC storage conditions, the decay or rotting values for treated samples are 17.08%, 27.87%, 39.6%, and 54.35%; 9.33%, 14.98%, 23.98%, and 36.07%; and 23.83%, 31.0%, 49.88%, and 66.38% for hot water‐treated, CaCl_2_‐treated, and control samples on the 8th, 15th, 22nd, and 29th days of storage, respectively, as shown in Table 3.

**Table 3:** Interaction effect of prestorage treatments and zero‐energy cooling chamber storage on decay or rotting of OFSP tuber.

Prestorage treatment	Storage condition	Storage days			
		8	15	22	29
Control		23.83 ± 1.62^a^	31.0 ± 0.56^a^	49.88 ± 1.08^a^	66.38 ± 1.02^a^
Hot water	ZECC	17.08 ± 0.98^b^	27.87 ± 056^b^	39.6 ± 2.03^b^	54.35 ± 1.53^b^
CaCl_2_		9.33 ± 2.47^c^	14.98 ± 2.07^c^	23.98 ± 1.43^c^	36.07 ± 1.02^c^
LSD		0.49	1.06	2.35	3.43
CV		3.90	3.34	2.71	1.50

*Note:* Means within the same column with different letters are significantly different (*p* < 0.05).

As shown in Table 4, calcium salt treatment effectively reduces decay and rotting of OFSP during storage in a ZECC by strengthening structural barriers, preserving membrane integrity, and delaying physiological senescence that predisposes tissues to pathogen invasion [30]. After harvest, OFSP tubers remain metabolically active and gradually undergo membrane deterioration, cell wall degradation, and moisture loss, creating favorable conditions for infection by postharvest pathogens such as *Fusarium*, *Rhizopus*, *Aspergillus*, *Penicillium*, and soft rot‐causing bacteria [31]. Calcium salts, particularly calcium chloride (CaCl_2_) and calcium lactate, penetrate the outer tissues and bind with de‐esterified pectic polysaccharides in the middle lamella to form calcium pectate cross‐links through the well‐established “egg‐box” model. This process reinforces the cell wall architecture, increases tissue firmness, and strengthens the mechanical barrier against pathogen penetration. Calcium also stabilizes plasma membranes by interacting with negatively charged phospholipid head groups, reducing membrane permeability and electrolyte leakage that would otherwise provide nutrients for microbial growth. In combination with the reduced temperature and high relative humidity maintained in a ZECC, these structural improvements delay tissue breakdown and substantially decrease the susceptibility of OFSP tubers to decay throughout storage [32].

**Table 4:** ANOVA of decay or rotting.

Source	DF	SS	Mean square	*F*	*Pr* > *F*
Pretreatments	2	2853.99	1426.99	376.50	< 0.0001
Storage_day	4	1341.05	335.26	88.46	< 0.0001
Storage_method	1	190.13	190.13	50.16	< 0.0001
Storage D*pretre*storage M	6	56.64	44.01	11.61	< 0.0001
Error	54	204.67	3.79		
Corrected total	80	5342.32			

*Note:* The asterisks denote the abbreviations for "D" (day) and "pretre" (pretreatment).

Beyond its structural role, calcium enhances the natural defense responses of OFSP by regulating physiological and biochemical processes that suppress disease development. Calcium acts as a secondary messenger in plant defense signaling pathways, activating the synthesis of defense‐related proteins and enzymes such as phenylalanine ammonia‐lyase (PAL), polyphenol oxidase (PPO), peroxidase (POD), chitinases, and *β*‐1,3‐glucanases. These enzymes promote lignification, phenolic compound accumulation, and strengthening of cell walls around infection sites, creating an unfavorable environment for pathogen colonization [33]. Calcium also stimulates antioxidant enzymes, including superoxide dismutase (SOD), catalase (CAT), and ascorbate peroxidase (APX), which scavenge reactive oxygen species (ROS) generated during storage stress. By reducing oxidative damage and suppressing membrane lipid peroxidation, calcium lowers malondialdehyde (MDA) accumulation and maintains cellular integrity. Furthermore, calcium inhibits the activities of cell wall‐degrading enzymes such as polygalacturonase, pectin methylesterase, cellulase, and lipoxygenase (LOX) that normally accelerate tissue maceration during senescence. The resulting maintenance of intact cell walls and membranes limits nutrient leakage into intercellular spaces, depriving pathogenic microorganisms of the readily available substrates required for rapid growth and disease progression [34].

The effectiveness of calcium treatment is further enhanced by the favorable storage environment provided by the ZECC, which operates through evaporative cooling to maintain temperatures of approximately 10°C–18°C and relative humidity of 85%–95% without the use of electricity. The lower storage temperature suppresses pathogen growth rates, slows respiration, and delays physiological aging, whereas the high relative humidity minimizes excessive moisture loss and prevents surface cracking that could facilitate microbial entry [35]. Calcium complements these environmental conditions by providing internal physiological protection through membrane stabilization, cell wall reinforcement, reduced respiration, improved water retention, and delayed senescence. Therefore, calcium‐treated OFSP tubers stored in a ZECC show significantly lower decay incidence and severity, reduced rotting, less tissue softening, and decreased PWL. They also maintain their visual appearance, firmness, and nutritional quality for a longer period compared with untreated tubers [36]. By integrating structural reinforcement with enhanced biochemical defense mechanisms and the naturally cooled ZECC environment, calcium salt treatment provides a practical, low‐cost, and environmentally sustainable postharvest technology for minimizing microbial spoilage, extending shelf life, and preserving the marketability of OFSP in regions where conventional refrigerated storage is unavailable [37].

### 3.4. Marketability

Under ZECC storage conditions as shown in Table 5, the values were 82.92%, 72.11%, 62.40%, and 59.65%; 89.67%, 80.02%, 69.02%, and 63.93%; and 82.17%, 69.00%, 60.12%, and 55.62% for hot water‐treated, CaCl_2_‐treated, and control samples on the 8th, 15th, 22nd, and 29th days of storage, respectively.

**Table 5:** Interaction effect of prestorage treatments and zero‐energy cooling chamber storage on marketability (%) of OFSP tuber.

Prestorage treatment	Storage condition	Storage days			
		8	15	22	29
Control		82.17 ± 0.76^c^	69.00 ± 2.99^c^	60.12 ± 1.30^b^	55.62 ± 1.88^c^
Hot water	ZECC	82.92 ± 0.95^c^	72.11 ± 2.03^b^	62.40 ± 1.77^b^	59.65 ± 2.18^b^
CaCl_2_		89.67 ± 0.76^a^	80.02 ± 1.65^a^	69.02 ± 2.52^a^	63.93 ± 1.37^a^
LSD		1.52	3.35	3.30	3.67
CV		1.02	2.67	3.27	3.08

*Note:* Means within the same column with different letters are significantly different (*p* < 0.05).

CaCl_2_‐treated OFSP tubers recorded the highest value. The prestorage treatments, storage conditions, and duration of storage significantly (*p* < 0.05) affect the marketability percentage, as shown in Table 6. Increasing CaCl_2_ concentrations improves marketability by inhibiting respiration, ethylene production, translation, and microbial growth [38]. Low‐temperature storage extends the shelf life of fruits and vegetables and minimizes PHLs by preventing decay [7].

**Table 6:** ANOVA of marketability.

Source	DF	SS	Mean square	*F*	*Pr* > *F*
Pretreatments	2	1058.82	529.41	208.39	< 0.0001
Storage_day	4	15,964.69	3991.17	1571.05	< 0.0001
Storage_method	1	354.27	354.27	139.45	< 0.0001
Storage D*pretre*storage M	6	124.93	20.82	8.20	< 0.0001
Error	54	137.18	2.54		
Corrected total	80	18,056.97			

*Note:* The asterisks denote the abbreviations for "D" (day) and "pretre" (pretreatment).

Calcium salt treatment plays a pivotal role in maintaining the marketability of OFSP during storage in a ZECC by preserving membrane integrity, delaying physiological deterioration, and sustaining the visual, nutritional, and textural attributes that determine consumer acceptance [39]. A ZECC typically operates through evaporative cooling, maintaining temperatures of approximately 10°C–18°C and relative humidity of 85%–95%, thereby reducing respiration, transpiration, and moisture loss. Although these conditions significantly slow postharvest deterioration, storage stress still induces membrane destabilization through oxidative damage, lipid peroxidation, and enzymatic degradation [40]. Application of calcium salts, most commonly calcium chloride (CaCl_2_) or calcium lactate, provides an additional physiological defense by stabilizing cellular membranes and cell walls, thereby complementing the protective environment created by the ZECC. The combined effects of calcium treatment and evaporative cooling preserve tissue integrity and prolong the commercial shelf life of OFSP, allowing roots to remain visually attractive, nutritionally valuable, and acceptable for marketing over extended storage periods [34].

The fundamental mechanism underlying calcium‐mediated membrane stabilization is the interaction of Ca^2+^ ions with negatively charged phospholipid head groups in the plasma membrane. During storage, oxidative stress causes membrane phospholipids to become disorganized, increasing membrane fluidity and permeability. This structural disruption results in leakage of cellular electrolytes, sugars, amino acids, organic acids, and vitamins, all of which accelerate tissue deterioration and quality loss [41]. Calcium ions form electrostatic bridges between adjacent phospholipid molecules, reducing electrostatic repulsion and increasing membrane cohesion. This stabilizing effect decreases membrane permeability and maintains selective transport across the membrane, thereby preserving cellular compartmentalization. Consequently, calcium‐treated OFSP roots exhibit significantly lower electrolyte leakage and higher membrane stability indices than untreated roots, reflecting greater preservation of membrane integrity throughout storage. Maintenance of membrane function directly contributes to sustained firmness, reduced tissue collapse, and delayed visible signs of senescence, which are essential attributes influencing consumer purchasing decisions [42].

Calcium also reinforces the structural stability of the cell wall–membrane complex through the formation of calcium pectate cross‐links within the middle lamella. The divalent calcium ions bind to free carboxyl groups of homogalacturonan chains in pectin according to the “egg‐box” model, producing a highly interconnected pectic network that strengthens adhesion between adjacent cells. This reinforced cell wall provides mechanical support to the plasma membrane and increases tissue resistance to compression, bruising, and handling damage during storage and transportation [43]. In OFSP, where postharvest softening results primarily from pectin depolymerization and middle lamella degradation, calcium‐induced cross‐linking markedly delays tissue softening and maintains root firmness. The retention of firmness preserves the characteristic appearance and texture expected by consumers and minimizes the incidence of shriveled, collapsed, or mechanically damaged roots that are often rejected in fresh produce markets [44].

### 3.5. Beta‐Carotene

Table 7 shows in ZECC, the values obtained were 50.18, 50.25, 49.32, and 48.56 mg/100 g; 55.79, 54.39, 54.12, and 53.27 mg/100 g; and 46.53, 46.09, 45.08, and 44.12 mg/100 g for hot‐water and Cacl_2_‐treated samples, as well as control samples, on the 8th, 15th, 22nd, and 29th days of storage time, respectively. These values gradually decreased as the storage days increased.

**Table 7:** Interaction effect of prestorage treatments and zero‐energy cooling chamber storage on beta‐carotene content (mg/100 g) of OFSP tuber.

Prestorage treatment	Storage condition	Storage days			
		8	15	22	29
Control		46.53 ± 1.62^c^	46.09 ± 0.56^c^	45.08 ± 1.08^c^	44.12 ± 1.03^c^
Hot water	ZECC	50.18 ± 0.98^b^	50.25 ± 056^b^	49.32 ± 2.03^b^	48.56 ± 1.53^b^
CaCl_2_		55.79 ± 2.47^a^	54.39 ± 2.07^a^	54.12 ± 1.43^a^	53.27 ± 1.02^a^
LSD		3.49	2.06	2.35	2.43
CV		3.90	2.34	2.71	2.50

*Note:* Means within the same column with different letters are significantly different (*p* < 0.05).

Prestorage treatments significantly impacted beta‐carotene levels throughout storage, regardless of whether ambient or ZECC conditions were used, as shown in Table 8, though the storage conditions themselves did not yield significant differences over time. At all storage times, all values were statistically (*p* < 0.05) different from one another. This demonstrated that the prestorage treatment significantly (p < 0.05) affected the beta‐carotene content of the OFSP sample. The beta‐carotene content decreases as the storage days increase. These values were consistent with [42], who reported that the beta‐carotene content of the OFSP sample fell within the range of 8.3–156.6 mg/100 g. Additionally, these values were closer to those reported by Ndanyi [45].

**Table 8:** ANOVA of beta‐carotene.

Source	DF	SS	Mean square	*F*	*Pr* > *F*
Pretreatments	2	1109.55	554.77	279.77	< 0.0001
Storage_day	4	102.26	25.57	12.89	< 0.0001
Storage_method	1	28.70	28.80	14.52	0.0004
Storage D*pretre*storage M	6	42.61	30.92	15.62	< 0.0001
Error	54	107.08	1.98		
Corrected total	80	1396.95			

*Note:* The asterisks denote the abbreviations for "D" (day) and "pretre" (pretreatment).

Calcium salt treatment plays a critical role in maintaining *β*‐carotene levels in OFSP during storage in a ZECC by preserving cellular integrity, reducing oxidative degradation, and delaying physiological senescence. *β*‐Carotene is primarily localized within chromoplasts, where it is highly susceptible to oxidation by ROS generated during postharvest respiration and storage stress [46]. Although the ZECC provides a favorable storage environment by maintaining temperatures of approximately 10°C–18°C and relative humidity of 85%–95%, oxidative metabolism continues throughout storage, gradually degrading carotenoid pigments. Calcium salts, such as calcium chloride (CaCl_2_) or calcium lactate, stabilize plasma membranes and chromoplast membranes through electrostatic interactions with phospholipid head groups while simultaneously strengthening cell walls by forming calcium pectate cross‐links within the middle lamella. This preservation of membrane integrity maintains the compartmentalization of *β*‐carotene within chromoplasts and prevents the mixing of carotenoids with oxidative enzymes, including LOX and POD, that accelerate pigment degradation [47]. Furthermore, calcium suppresses membrane lipid peroxidation by reducing MDA accumulation, thereby protecting the lipid‐rich chromoplast membranes where *β*‐carotene is stored. These structural and biochemical effects significantly slow carotenoid oxidation, allowing calcium‐treated OFSP roots to retain their characteristic deep orange flesh color and higher provitamin A activity during extended storage [48].

In addition to membrane stabilization, calcium enhances the antioxidant defense system and moderates respiratory metabolism, both of which contribute substantially to *β*‐carotene preservation [49]. Calcium functions as an intracellular signaling molecule that activates antioxidant enzymes such as SOD, CAT, APX, and POD, which collectively scavenge superoxide radicals, hydrogen peroxide, and hydroxyl radicals before they can oxidize carotenoid molecules. At the same time, calcium stabilizes mitochondrial membranes and reduces respiration rates, thereby lowering oxygen consumption and minimizing the production of ROS associated with oxidative metabolism [50]. The cooler, high‐humidity environment of the ZECC further complements these effects by slowing enzymatic activity, reducing metabolic stress, and limiting moisture loss, all of which contribute to delayed senescence. Consequently, calcium‐treated OFSP roots stored in a ZECC exhibit greater *β*‐carotene retention, improved antioxidant capacity, enhanced nutritional quality, and prolonged maintenance of the bright orange pigmentation that is closely associated with consumer acceptance and the nutritional value of this biofortified crop [51].

### 3.6. Vitamin C

For the hot water‐treated, CaCl_2_‐treated, and control samples on the 8th, 15th, 22nd, and 29th days of storage, the vitamin C content of the OFSP samples examined in ZECC was 6.77, 6.47, 6.25, and 5.96 mg/100 g; 7.89, 7.67, 7.38, and 7.15 mg/100 g; and 7.11, 6.64, 6.54, and 6.38 mg/100 g, respectively. These values were consistent with findings that ranged from 7.61 to 16.79 mg/100 g for the vitamin C content in OFSP samples, as shown in Table 9.

**Table 9:** Interaction effect of prestorage treatments and zero‐energy cooling chamber storage on vitamin C content (mg/100 g) of OFSP tubers.

Prestorage treatment	Storage condition	Storage days			
		8	15	22	29
Control		7.11 ± 0.15^b^	6.64 ± 0.18^b^	6.54 ± 0.25b	6.38 ± 0.02^b^
Hot water	ZECC	6.77 ± 0.21^c^	6.47 ± 0.20^b^	6.25 ± 0.10^c^	5.96 ± 0.10^c^
CaCl_2_		7.89 ± 0.01^a^	7.67 ± 0.21^a^	7.38 ± 0.15^a^	7.15 ± 0.02^a^
LSD		0.30	0.39	0.33	0.38
CV		2.34	3.22	2.84	2.94

*Note:* Means within the same column that have different letters are significantly different (*p* < 0.05).

CaCl_2_‐treated samples effectively preserved vitamin C in OFSP under ambient and ZECC conditions throughout storage. In ZECC, these samples showed no significant difference in vitamin C content, except on Day 29. The hot water‐treated samples exhibited significant differences (*p* < 0.05) throughout the storage days in both storage conditions, as shown in Table 10. The findings agreed with those Gharezi et al. [52], who reported that CaCl_2_‐treated tomatoes retained more vitamin C content than untreated (control) samples. Ascorbic acid levels progressively decline during storage and are particularly vulnerable to oxidation during food preparation and storage, making it a critical indicator of overall nutrient quality. These findings demonstrate that CaCl_2_ treatments significantly impacted the retention of ascorbic acid content. This may result from greater CaCl_2_ concentrations delaying the rapid oxidation of ascorbic acid during storage [53].

**Table 10:** ANOVA of vitamin C.

Source	DF	SS	Mean square	*F*	*Pr* > *F*
Pretreatments	2	15.69	7.85	94.35	< 0.0001
Storage_day	4	11.14	2.79	33.50	< 0.0001
Storage_method	1	0.51	0.51	6.17	0.02
Storage D*pretre*storage M	6	1.17	0.83	10.33	< 0.0001
Error	54	4.49	0.08		
Corrected total	80	32.89			

*Note:* The asterisks denote the abbreviations for "D" (day) and "pretre" (pretreatment).

Calcium salt treatment plays a crucial role in retaining carotenoids and vitamin C in OFSP during storage in a ZECC by reducing oxidative stress, stabilizing cellular membranes, and slowing senescence‐related metabolic processes [33]. Carotenoids, particularly *β*‐carotene, and vitamin C are highly susceptible to degradation by ROS, enzymatic oxidation, and exposure to elevated temperatures. Although the ZECC provides a naturally cooled environment with temperatures typically ranging from 10°C to 18°C and relative humidity of 85%–95%, oxidative deterioration continues to occur during prolonged storage [54]. Calcium salts such as calcium chloride (CaCl_2_) or calcium lactate complement the protective effect of the ZECC by preserving membrane integrity and maintaining cellular compartmentalization. Calcium ions bind to phospholipid head groups in the plasma membrane and cross‐link pectic substances in the cell wall, reducing membrane permeability and preventing leakage of antioxidants and oxidative enzymes from intracellular compartments. This structural stabilization minimizes contact between carotenoids and pro‐oxidant enzymes such as LOX and POD, thereby reducing pigment oxidation and preserving the characteristic deep orange color of OFSP roots. Simultaneously, calcium suppresses membrane lipid peroxidation by lowering MDA accumulation, thereby maintaining the intracellular environment necessary for the stability of both *β*‐carotene and ascorbic acid throughout storage [55].

Calcium further enhances the retention of carotenoids and vitamin C by activating the endogenous antioxidant defense system and moderating respiratory metabolism. During postharvest storage, respiration continuously generates ROS, including superoxide radicals, hydrogen peroxide, and hydroxyl radicals, which readily oxidize carotenoids and convert ascorbic acid into dehydroascorbic acid, followed by irreversible degradation [56]. Calcium acts as a secondary messenger in cellular signaling pathways, stimulating the activities of antioxidant enzymes such as SOD, CAT, APX, glutathione reductase (GR), and POD. These enzymes efficiently detoxify ROS before they can attack membrane lipids and sensitive bioactive compounds [57]. In addition, calcium stabilizes mitochondrial membranes and reduces respiration rates, thereby lowering oxygen consumption and limiting oxidative metabolism during storage. The reduced respiratory activity conserves carbohydrate reserves and decreases the generation of free radicals responsible for nutrient degradation. Consequently, calcium‐treated OFSP roots exhibit significantly greater retention of *β*‐carotene, vitamin C, total antioxidant capacity, and overall nutritional quality than untreated roots stored under identical ZECC conditions [58].

The synergistic interaction between calcium treatment and the storage environment provided by the ZECC ultimately maximizes the preservation of nutritional quality and extends the marketable shelf life of OFSP [59]. The ZECC reduces temperature and maintains high relative humidity, slowing physiological processes, minimizing transpiration, and reducing moisture loss, whereas calcium provides internal biochemical protection by strengthening membrane and cell wall structures, suppressing oxidative damage, and delaying senescence [60]. These complementary mechanisms preserve chromoplast integrity, where carotenoids are stored, preventing pigment degradation and maintaining the bright orange flesh color that serves as an important indicator of provitamin A content and consumer acceptance. Likewise, the preservation of membrane integrity limits the oxidation of vitamin C by maintaining compartmentalization between ascorbic acid and oxidative enzymes such as ascorbate oxidase. As a result, calcium‐treated OFSP stored in a ZECC retains higher concentrations of *β*‐carotene and vitamin C, exhibits greater antioxidant activity, maintains superior visual quality and nutritional value, and experiences lower PHLs than untreated roots. This combined postharvest strategy therefore represents an effective, low‐cost, and sustainable approach for conserving the health‐promoting compounds of OFSP while ensuring prolonged storage stability and marketability in regions lacking conventional refrigerated storage facilities [61].

### 3.7. Proximate Composition

#### 3.7.1. Moisture Content

Under ZECC storage conditions, the values recorded were 83.1%, 81.4%, 79.7%, and 77.7%; 78.7%, 77.2%, 74.1%, and 72.3%; and 68.75, 68.0%, 65.0%, and 62.5% for hot water‐treated, CaCl_2_‐treated, and control samples on the 8th, 15th, 22nd, and 29th days of storage time, respectively, as shown in Table 11. This indicates that the moisture content decreases as storage time increases. The outcomes align with values of 80%, 78%, and 73% for blanched, CaCl_2_‐treated, and control samples, respectively, as reported by Dereje et al. [62]. Moisture content values consistently differed significantly based on prestorage treatment, indicating its substantial impact on OFSP tubers, as shown in Table 12, except on the 8th day when hot water‐treated tubers showed no significant difference whether stored ambiently or in a ZECC.

**Table 11:** Interaction effect of prestorage treatments and zero‐energy cooling chamber storage on the proximate composition (%) of OFSP tuber.

Storage days	Prestorage treatment	Storage condition	Moisture content	Crude protein	Crude fat	Crude fiber	Ash content	Utilizable carbohydrate
8	Control	ZECC	68.7 ± 0.27^d^	5.07 ± 0.16^b^	1.32 ± 0.04^a^	2.43 ± 0.02^d^	2.65 ± 0.02^c^	81.77 ± 0.14^a^
	Hot water		83.1 ± 0.17^a^	4.57 ± 0.29^c^	1.19 ± 0.01^c^	2.50 ± 0.02^c^	2.68 ± 0.02^c^	80.77 ± 0.42^b^
	CaCl_2_		78.7 ± 0.23^b^	6.28 ± 0.22^a^	1.22 ± 0.03^c^	2.65 ± 0.04^a^	2.94 ± 0.01^b^	79.04 ± 0.45^d^

15	Control	ZECC	68.0 ± 0.08^d^	4.47 ± 0.27^c^	1.24 ± 0.07^a^	2.37 ± 0.06^b^	2.67 ± 0.01^e^	81.92 ± 0.13^b^
	Hot water		81.4 ± 0.02^a^	4.47 ± 0.27^c^	1.04 ± 0.06^b^	2.38 ± 0.05^b^	2.75 ± 0.04^d^	81.22 ± 0.26^c^
	CaCl_2_		77.2 ± 0.09^b^	6.19 ± 0.06^a^	1.21 ± 0.02^a^	2.57 ± 0.04^a^	2.98 ± 0.01^b^	79.34 ± 0.17^e^

22	Control	ZECC	65.0 ± 0.01^e^	4.33 ± 0.20^d^	1.15 ± 0.07^a^	2.23 ± 0.01^c^	2.70 ± 0.01^e^	82.55 ± 0.03^b^
	Hot water		79.7 ± 0.02^a^	4.33 ± 0.20^d^	0.99 ± 0.05^a^	2.31 ± 0.02^b^	2.93 ± 0.01^d^	81.49 ± 0.19^c^
	CaCl_2_		74.1 ± 0.05^b^	6.05 ± 0.06^a^	1.10 ± 0.02^a^	2.45 ± 0.02^a^	3.11 ± 0.09^b^	79.90 ± 0.11^d^

29	Control	ZECC	62.5 ± 0.14^c^	4.72 ± 0.11^b^	0.98 ± 0.02^a^	2.19 ± 0.04^b^	2.73 ± 0.02c	83.13 ± 0.26^a^
	Hot water		77.7 ± 0.03^a^	4.13 ± 0.11^c^	0.85 ± 0.06^b^	2.24 ± 0.03^b^	2.96 ± 0.05b	82.05 ± 0.13^b^
	CaCl_2_		72.3 ± 0.05^b^	5.85 ± 0.12^a^	0.92 ± 0.05^a^	2.36 ± 0.06^a^	3.24 ± 0.04a	80.41 ± 0.24^c^

LSD			0.17	0.23	0.09	0.09	0.07	0.42

CV			1.22	2.30	4.93	1.93	1.31	0.27

*Note:* Means within the same column with different letters are significantly different (*p* < 0.05).

**Table 12:** ANOVA of moisture content.

Source	DF	SS	Mean square	*F*	*Pr* > *F*
Pretreatments	2	24.70	12.35	571.13	< 0.0001
Storage_day	4	14.12	3.53	163.25	< 0.0001
Storage_method	1	4.36	4.36	201.46	< 0.0001
Storage D*pretre*storage M	6	0.32	0.05	2.49	0.0337
Error	54	1.17	0.02		
Corrected total	80	46.71			

*Note:* The asterisks denote the abbreviations for "D" (day) and "pretre" (pretreatment).

Calcium salt treatment significantly reduces moisture migration in OFSP during storage in a ZECC by maintaining the structural integrity of cellular membranes and reinforcing the cell wall matrix [42]. Moisture migration in harvested storage roots is primarily driven by water vapor pressure gradients between the tissue and the surrounding environment, coupled with membrane deterioration and cell wall degradation that increase cellular permeability. Although the ZECC maintains a favorable storage environment with relatively low temperatures (approximately 10°C–18°C) and high relative humidity (85%–95%), physiological water loss cannot be completely eliminated during prolonged storage [33]. Calcium salts, particularly calcium chloride (CaCl_2_) and calcium lactate, provide an additional protective mechanism by binding to negatively charged phospholipid head groups in the plasma membrane, thereby stabilizing membrane structure and reducing membrane permeability. Simultaneously, calcium cross‐links pectic polysaccharides within the middle lamella through the formation of calcium pectate (egg‐box) structures, strengthening cell walls and preserving cell‐to‐cell adhesion. These structural modifications reduce the diffusion of intracellular water into intercellular spaces and ultimately decrease transpiration from the root surface. Consequently, calcium‐treated OFSP roots maintain higher tissue moisture content, experience lower PWL, and exhibit reduced surface shriveling compared with untreated roots throughout storage [63].

In addition to its structural effects, calcium regulates cellular water homeostasis by maintaining membrane selectivity and preserving the function of membrane‐bound transport proteins involved in water movement. During storage, senescence‐induced membrane deterioration disrupts osmotic regulation, causing uncontrolled leakage of water and dissolved solutes from vacuoles and the cytoplasm into extracellular spaces [64]. Calcium stabilizes plasma membranes and tonoplast membranes, maintaining the integrity of osmotic gradients that govern intracellular water distribution. Furthermore, calcium influences the activity of aquaporins, specialized membrane proteins that facilitate regulated water transport across cellular membranes. By preserving aquaporin functionality and preventing membrane lipid peroxidation, calcium enables controlled water movement while minimizing passive water loss from storage tissues [65]. At the same time, calcium suppresses the activities of phospholipase D (PLD) and LOX, enzymes responsible for membrane phospholipid degradation, thereby preventing membrane disruption that would otherwise accelerate moisture migration. The reduced oxidative damage also lowers MDA accumulation, an indicator of lipid peroxidation, ensuring that membrane permeability remains low throughout storage. These physiological mechanisms collectively enable calcium‐treated roots to retain cellular turgor pressure, maintain firmness, and preserve the fresh appearance essential for prolonged storage [66].

The interaction between calcium salt treatment and the microclimatic conditions provided by the ZECC creates a synergistic system for minimizing moisture migration and extending the storage life of OFSP [67]. The ZECC lowers storage temperature through evaporative cooling while maintaining a high‐humidity environment that decreases the vapor pressure deficit between the root surface and the surrounding air, thereby reducing transpiration rates. Calcium complements these environmental conditions by providing internal physiological protection through membrane stabilization, strengthened cell walls, improved osmotic regulation, and delayed senescence [68]. Reduced respiration and metabolic activity further conserve intracellular water by decreasing the breakdown of structural carbohydrates and limiting the formation of microscopic tissue cracks that facilitate water escape. As a result, calcium‐treated OFSP roots stored in a ZECC maintain higher moisture content, greater tissue firmness, smoother skin, and improved visual quality throughout extended storage. The retention of moisture not only preserves fresh weight and minimizes economic losses but also supports the maintenance of texture, nutritional quality, and consumer acceptability, making the combined use of calcium salt treatment and ZECCs an effective, low‐cost, and sustainable postharvest technology for extending the shelf life and marketability of OFSP [69].

The findings were in line with Dereje et al. [62] and Adam et al. [70] those who reported that compared with controls, both blanched and CaCl_2_‐treated samples exhibited higher moisture content. However, an optimal CaCl_2_ concentration reduced moisture, possibly because increased Ca^2+^ ions promoted more calcium‐pectate complex formation [70]. The moisture content obtained fell within an acceptable range for flour [71], remaining below 14%. Flour with less than 14% moisture content is shelf‐stable due to its resistance to microbial growth. Maintaining appropriate moisture levels is crucial for flour storage to prevent quality degradation and loss of functionality [72].

#### 3.7.2. Crude Protein Content

Under ZECC storage conditions, the crude protein content values of OFSP tubers were 4.13%, 5.85%, and 4.72% for the hot water‐treated, CaCl_2_‐treated, and control samples on the 29th day, respectively, as shown in Table 11. Prestorage treatments significantly alter the protein content of food products; notably, CaCl_2_‐treated samples consistently showed the highest protein values across all storage days when compared with hot water‐treated and untreated samples. These results demonstrate a statistically significant difference (*p* < 0.05) between the storage conditions (ambient and ZECC) and the storage days, as shown in Table 13. Additionally, Panchal et al. [73] reported that hot water‐blanched sweet potato tubers had lower protein content than samples treated with CaCl_2_. Thus, the combination of CaCl_2_ prestorage treatment and ZECC storage effectively retained protein content during the storage period.

**Table 13:** ANOVA of crude protein.

Source	DF	SS	Mean square	*F*	*Pr* > *F*
Pretreatments	2	41.40	20.70	729.47	< 0.0001
Storage_day	4	4.84	1.21	42.63	< 0.0001
Storage_method	1	0.42	0.42	14.62	0.0003
Storage D*pretre*storage M	6	0.58	0.34	11.45	< 0.0001
Error	54	1.53	0.03		
Corrected total	80	50.07			

*Note:* The asterisks denote the abbreviations for "D" (day) and "pretre" (pretreatment).

Calcium salt treatment contributes to the maintenance of crude protein content in OFSP during storage in a ZECC by preserving cellular integrity, delaying senescence, and reducing protein degradation associated with postharvest metabolism [63]. Following harvest, storage roots remain physiologically active and continue to respire, leading to the gradual breakdown of proteins into amino acids that are utilized as substrates for respiration and stress responses. Calcium salts, particularly calcium chloride (CaCl_2_) and calcium lactate, stabilize plasma membranes by binding to negatively charged phospholipids and strengthen cell walls through the formation of calcium pectate cross‐links. These structural effects maintain membrane compartmentalization, preventing the leakage of intracellular proteins and reducing the interaction between proteins and proteolytic enzymes [71]. Calcium also suppresses membrane lipid peroxidation by lowering ROS accumulation and MDA formation, thereby protecting protein molecules from oxidative modification and denaturation. Furthermore, calcium delays physiological aging by reducing respiration rates and metabolic activity, conserving nitrogen‐containing compounds and slowing the conversion of storage proteins into respiratory substrates. Consequently, calcium‐treated OFSP roots retain higher crude protein content than untreated tubers during prolonged storage [69].

The preservation of crude protein is further enhanced by the synergistic interaction between calcium treatment and the storage conditions provided by the ZECC [66]. The ZECC maintains a relatively low temperature (approximately 10°C–18°C) and high relative humidity (85%–95%), which collectively reduce respiration, enzyme activity, and moisture loss while delaying tissue senescence. Calcium complements these favorable environmental conditions by inhibiting the activity of proteolytic enzymes responsible for protein hydrolysis and by activating antioxidant defense enzymes such as SOD, CAT, and APX, which minimize oxidative stress that can damage proteins [74]. Reduced oxidative deterioration preserves the structural and functional integrity of cellular proteins and limits nitrogen losses during storage. Additionally, the maintenance of membrane integrity and tissue hydration reduces cellular stress and decreases the demand for protein catabolism as an alternative energy source. As a result, calcium‐treated OFSP stored in a ZECC exhibits improved crude protein retention, greater nutritional quality, enhanced tissue integrity, and prolonged shelf life compared with untreated roots, demonstrating the effectiveness of integrating calcium salt treatment with evaporative cooling technology for sustainable postharvest preservation [64].

#### 3.7.3. Crude Fat Content

In ZECC storage conditions, the values were 1.19%, 1.04%, 0.99%, and 0.85%; 1.22%, 1.21%, 1.10%, and 0.92%; and 1.32%, 1.24%, 1.15%, and 0.98% for hot‐water treated, CaCl_2_‐treated, and untreated (control) samples, respectively, on Days 8, 15, 22, and 29 of storage, as shown in Table 11, which were in agreement with Abrham et al. [75]. The fat content of the OFSP sample decreased as the storage days increased. This indicates that the fat content of the OFSP sample was significantly (*p* < 0.05) affected by the storage duration and prestorage treatments, as shown in Table 14. A slight difference, though not significant, was observed between the storage conditions. Fat depletion in hot‐water samples may be attributed to oxidation as the temperature rises [75].

**Table 14:** ANOVA of crude fat.

Source	DF	SS	Mean square	*F*	*Pr* > *F*
Pretreatments	2	0.26	0.13	36.88	< 0.0001
Storage_day	4	1.12	0.28	78.71	< 0.0001
Storage_method	1	0.02	0.02	5.00	0.03
Storage D*pretre*storage M	6	1.001	0.683	17.09	< 0.0001
Error	54	0.19	0.004		
Corrected total	80	1.65			

*Note:* The asterisks denote the abbreviations for "D" (day) and "pretre" (pretreatment).

Calcium salt treatment helps maintain the crude fat content of OFSP during storage in a ZECC by protecting membrane lipids from oxidative degradation and preserving the structural integrity of lipid‐containing cellular organelles [65]. Although OFSP contains relatively low levels of crude fat, these lipids are physiologically important because they are major constituents of cellular and organelle membranes, particularly the chromoplast membranes that store *β*‐carotene. During postharvest storage, ROS generated through respiration initiate lipid peroxidation, leading to the oxidation of unsaturated fatty acids, disruption of membrane phospholipids, and gradual loss of extractable crude fat [76]. Calcium salts, such as calcium chloride (CaCl_2_) or calcium lactate, stabilize plasma membranes by binding to negatively charged phospholipid head groups, thereby reducing membrane permeability and increasing membrane rigidity. Calcium also suppresses the activities of lipid‐degrading enzymes, particularly PLD and LOX, which catalyze phospholipid hydrolysis and oxidation of unsaturated fatty acids during senescence. In addition, calcium enhances the activities of antioxidant enzymes including SOD, CAT, and APX, reducing ROS accumulation and MDA formation, a key indicator of lipid peroxidation. These combined mechanisms preserve membrane phospholipids and minimize oxidative degradation, thereby maintaining crude fat levels throughout storage [77].

The preservation of crude fat is further enhanced by the synergistic interaction between calcium treatment and the favorable microenvironment provided by the ZECC. The ZECC maintains temperatures of approximately 10°C–18°C and relative humidity of 85%–95%, conditions that slow respiration, reduce metabolic activity, and limit oxidative stress while minimizing moisture loss [77]. Calcium complements these storage conditions by delaying physiological senescence, preserving membrane integrity, and maintaining cellular compartmentalization, thereby reducing the metabolic demand for membrane lipid turnover. Lower respiration rates decrease oxygen consumption and the generation of free radicals that accelerate lipid oxidation, whereas improved tissue hydration minimizes membrane disruption associated with cellular dehydration [78]. Furthermore, the preservation of chromoplast membrane integrity helps maintain the lipid matrix that protects *β*‐carotene, contributing simultaneously to improved carotenoid stability and crude fat retention. Consequently, calcium‐treated OFSP stored in a ZECC generally exhibits lower lipid oxidation, reduced membrane deterioration, improved nutritional quality, and greater retention of crude fat than untreated roots. Although changes in crude fat content during storage are often modest because OFSP is naturally low in lipids, the combined effects of calcium‐induced membrane stabilization and ZECC storage effectively preserve the lipid fraction and contribute to prolonged shelf life and overall quality maintenance [78].

#### 3.7.4. Crude Fiber Content

The values for crude fiber content in ZECC were recorded as 2.50%, 2.38%, 2.31%, and 2.24%; 2.65%, 2.57%, 2.45%, and 2.36%; and 2.43%, 2.37%, 2.23%, and 2.19% for hot‐water‐treated, CaCl_2_‐treated, and control samples on the 8th, 15th, 22nd, and 29th days of storage, respectively, as shown in Table 11. This indicated a significant (*p* < 0.05) difference in the crude fiber content of the OFSP sample, as shown in Table 15. Additionally, the crude fiber content of OFSP samples decreased as the duration of storage increased.

**Table 15:** ANOVA of crude fiber.

Source	DF	SS	Mean square	*F*	*Pr* > *F*
Pretreatments	2	0.52	0.26	95.28	< 0.0001
Storage_day	4	1.41	0.35	130.76	< 0.0001
Storage_method	1	0.13	0.13	47.58	< 0.0001
Storage D*pretre*storage M	6	0.81	0.622	20.75	< 0.0001
Error	54	0.15	0.003		
Corrected total	80	2.26			

*Note:* The asterisks denote the abbreviations for "D" (day) and "pretre" (pretreatment).

Tubers pretreated with CaCl_2_ and stored in ZECC showed a significant difference from other treatments by Day 15, with overall prestorage treatment differences becoming notable by Day 22. The samples treated with CaCl_2_ in ZECC displayed the highest crude fiber content across all storage days. In contrast, the untreated (control) samples recorded the lowest values, with significant differences (*p* > 0.05) compared with the CaCl_2_‐treated samples in ZECC. These findings align with Ezeama et al. [72] and Ngoma et al. [71], who reported that samples treated with CaCl_2_ had higher fiber content than untreated (control) samples. Recently, dietary fiber has gained considerable importance as it is said to reduce the incidences of colon cancer, diabetes, heart disease, and certain digestive disorders [79].

Calcium salt treatment helps maintain the crude fiber content of OFSP during storage in a ZECC by preserving the structural integrity of the cell wall and reducing enzymatic degradation of fibrous polysaccharides [80]. Crude fiber in OFSP consists primarily of cellulose, hemicellulose, and lignin, which provide mechanical strength and contribute to the texture of the storage root. During postharvest storage, natural senescence activates cell wall‐degrading enzymes, including polygalacturonase, pectin methylesterase, cellulase, and hemicellulase, which progressively degrade structural polysaccharides, resulting in tissue softening and deterioration [81]. Calcium salts, particularly calcium chloride (CaCl_2_) and calcium lactate, reinforce the cell wall by forming calcium pectate cross‐links between de‐esterified pectin molecules in the middle lamella through the “egg‐box” mechanism. These cross‐links strengthen cell‐to‐cell adhesion, reduce cell wall porosity, and increase resistance to enzymatic hydrolysis. Calcium also suppresses the activities of cell wall‐degrading enzymes by maintaining membrane integrity and delaying senescence, thereby preserving the cellulose–hemicellulose network that contributes to crude fiber. Consequently, calcium‐treated OFSP roots retain greater structural firmness and experience less degradation of dietary fiber components during storage than untreated roots [82].

The preservation of crude fiber is further enhanced by the synergistic interaction between calcium treatment and the favorable storage conditions provided by the ZECC. The ZECC maintains temperatures of approximately 10°C–18°C and relative humidity of 85%–95%, which reduce respiration, slow metabolic activity, and delay physiological aging while minimizing excessive moisture loss [41]. Lower temperatures suppress the synthesis and activity of hydrolytic enzymes responsible for cell wall disassembly, whereas high relative humidity reduces dehydration‐induced structural stress that can accelerate tissue breakdown. Calcium complements these environmental effects by stabilizing plasma membranes, reducing oxidative stress through enhanced antioxidant enzyme activity, and preserving cellular compartmentalization, thereby limiting the release and activation of enzymes involved in fiber degradation [83]. The maintenance of cell wall architecture also reduces susceptibility to microbial invasion, as intact structural polysaccharides serve as an effective physical barrier against pathogen penetration. As a result, calcium‐treated OFSP stored in a ZECC exhibits greater retention of crude fiber, improved textural quality, enhanced firmness, delayed softening, and prolonged shelf life compared with untreated roots. Although crude fiber content generally changes only modestly during storage, the combined effects of calcium‐induced cell wall reinforcement and evaporative cooling effectively preserve the structural carbohydrate fraction and contribute to maintaining the overall quality and marketability of OFSP [84].

#### 3.7.5. Ash Content

In ZECC storage conditions, the recorded values were 2.68%, 2.75%, 2.93%, and 2.96%; 2.94%, 2.98%, 3.11%, and 3.24%; and 2.65%, 2.67%, 2.70%, and 2.73% for hot water‐treated, CaCl_2_‐treated, and control samples on the 8th, 15th, 22nd, and 29th days of storage, respectively, as shown in Table 11. Prestorage treatments notably influenced the ash content of the OFSP sample; specifically, CaCl_2_ treatment led to a higher ash content due to an increase in the product′s calcium content. On the 8th day of storage, the CaCl_2_‐treated OFSP sample recorded the highest value of 2.94% under ZECC storage conditions, which was significantly (*p* < 0.05) different from the other prestorage treatments. However, no significant (*p* > 0.05) difference was found between the hot water‐treated and untreated (control) samples.

The untreated (control) samples stored in ZECC on the 29th day of storage had the lowest value at 2.73%. Storage conditions had a significant (*p* < 0.05) effect on the ash content of the OFSP sample, as shown in Table 16. The ash content of the OFSP tuber increased with longer storage times; however, its value depends on prestorage treatments. The CaCl_2_‐treated samples under ambient conditions exhibited the highest value. This finding aligns with Adeoye et al. [85] and Agbemafle et al. [79] reports that the ash content increased as the number of storage days rose. The sweet potato samples treated with CaCl_2_ had the highest ash content, possibly due to increased calcium content [86].

**Table 16:** ANOVA of ash content.

Source	DF	SS	Mean square	*F*	*Pr* > *F*
Pretreatments	2	1.92	0.96	887.65	< 0.0001
Storage_day	4	0.63	0.16	146.24	< 0.0001
Storage_method	1	0.02	0.02	17.33	0.0001
Storage D*pretre*storage M	6	0.113	0.025	25.48	< 0.0001
Error	54	0.06	0.001		
Corrected total	80	2.72			

*Note:* The asterisks denote the abbreviations for "D" (day) and "pretre" (pretreatment).

Calcium salt treatment contributes to the maintenance of ash content in OFSP during storage in a ZECC by preserving cellular integrity and minimizing the loss of mineral constituents through membrane deterioration and tissue degradation [87]. Ash content represents the total inorganic mineral fraction of the storage root, including calcium, potassium, magnesium, phosphorus, sodium, iron, and other essential elements that are retained within the cellular matrix. During prolonged storage, senescence‐induced membrane damage increases membrane permeability, resulting in the leakage of mineral ions from vacuoles and the cytoplasm into intercellular spaces, where they may be lost through exudation or become associated with deteriorated tissues removed during decay [88]. Calcium salts, particularly calcium chloride (CaCl_2_) and calcium lactate, stabilize plasma membranes by interacting with negatively charged phospholipid head groups and reinforce cell walls through the formation of calcium pectate cross‐links in the middle lamella. These structural modifications maintain membrane selectivity, reduce electrolyte leakage, and preserve the intracellular localization of mineral nutrients. In addition, calcium delays physiological senescence by reducing respiration and oxidative stress, thereby maintaining normal membrane function and preventing excessive mineral redistribution or loss during storage. Consequently, calcium‐treated OFSP roots generally retain higher ash content and overall mineral quality than untreated tubers throughout the storage period [89].

The preservation of ash content is further enhanced by the synergistic effects of calcium treatment and the favorable storage environment provided by the ZECC. The ZECC maintains temperatures of approximately 10°C–18°C and relative humidity of 85%–95%, conditions that reduce metabolic activity, minimize transpiration, and slow tissue deterioration without the use of electrical refrigeration [90]. Lower respiration rates decrease the turnover of cellular components, whereas high relative humidity limits excessive moisture loss that could otherwise concentrate solutes and promote membrane disruption. Calcium complements these environmental benefits by activating antioxidant defense systems that reduce ROS accumulation and membrane lipid peroxidation, thereby preserving the integrity of ion transport systems and cellular compartmentalization. Furthermore, the strengthened cell wall–membrane complex decreases susceptibility to microbial infection and tissue maceration, reducing mineral losses associated with decay and rotting. It is important to note that ash content is generally one of the more stable proximate components during storage; therefore, any observed improvements are primarily attributable to enhanced mineral retention through reduced physiological deterioration rather than an actual increase in mineral concentration. The combined use of calcium salt treatment and ZECC storage thus provides an effective and sustainable strategy for maintaining the mineral composition, nutritional quality, and overall postharvest stability of OFSP [91].

#### 3.7.6. Utilizable Carbohydrate

In ZECC, the values obtained were 81.77%, 81.92%, 82.55%, and 83.13% for control samples, and 80.77%, 81.22%, 81.49%, and 82.05%; 79.04%, 79.34%, 79.90%, and 80.41% for hot‐water and CaCl_2_‐treated samples on the 8th, 15th, 22nd, and 29th days of storage, respectively, as shown in Table 11. The utilizable carbohydrate content of the OFSP tuber was significantly (*p* < 0.05) affected by the storage day, as shown in Table 17. In terms of prestorage treatments, the untreated (control) OFSP tuber exhibited the highest level of utilizable carbohydrates compared with the hot‐water and CaCl_2_‐treated samples. Observations indicated a percentage increase in carbohydrate composition, consistent with Rickman et al. [92], who found that the storage period influences this increase.

**Table 17:** ANOVA of utilizable carbohydrates.

Source	DF	SS	Mean square	*F*	*Pr* > *F*
Pretreatments	2	86.19	43.10	500.99	< 0.0001
Storage_day	4	53.01	13.25	154.05	< 0.0001
Storage_method	1	9.53	9.53	110.73	< 0.0001
Storage D*pretre*storage M	6	2.63	1.91	21.21	< 0.0001
Error	54	4.65	0.09		
Corrected total	80	158.29			

*Note:* The asterisks denote the abbreviations for "D" (day) and "pretre" (pretreatment).

## 4. Conclusions

Optimizing prestorage treatments and storage conditions significantly improved tuber marketability and reduced PWL. The use of calcium chloride treatment combined with ZECC enhanced the physicochemical properties and shelf life of OFSP tubers. Specifically, calcium chloride pretreatments and ZECC storage preserved tuber quality and extended storage longevity. OFSP tubers treated with CaCl_2_ retained higher levels of ascorbic acid and beta‐carotene compared with hot water‐treated and control samples. Furthermore, CaCl_2_‐treated tubers stored in ZECC exhibited the lowest weight loss (9.17%) and the highest marketability (63.93%), while maintaining 53.27 mg/100 g of beta‐carotene and 7.15 mg/100 g of vitamin C. The proximate composition of CaCl_2_‐treated OFSP tubers was 80.41% available carbohydrate, 72.3% moisture, 5.85% crude protein, 3.24% ash, 2.36% crude fiber, and 0.92% crude fat. Calcium chloride prestorage treatment benefits sweet potato tubers by retaining quality, reducing waste, and extending shelf life. Farmers in developing nations like Ethiopia can effectively preserve OFSP by combining calcium chloride pretreatment with ZECC storage. Future research should focus on the mineral, microbial analysis and phytochemical characteristics of orange‐fleshed produce.

## Author Contributions

**Derartu Girma Deressa:** conceptualization, methodology, formal analysis, data curation, investigation, writing – original draft, visualization, validation. **Getachew Neme Tolesa:** data curation, methodology, validation, writing – review and editing, supervision. **Solomon Abera Habtegebriel:** data curation, methodology, validation, writing – review and editing, supervision. **Fekireselassia Mekuria Mesfine:** data curation, validation, writing – review and editing, supervision. **Dessalegn Legese Negewo:** data curation, validation, writing – review and editing, supervision. **Alemayehu Hirko Muleta:** validation, writing – review and editing, supervision. **Abdeta Tadesse Adugna:** validation, writing – review and editing, supervision.

## Funding

No funding was received for this manuscript.

## Disclosure

All authors have reviewed and approved the final version of the manuscript.

## Ethics Statement

All experiments adhered to established ethical guidelines of Haramaya University.

## Conflicts of Interest

The authors declare no conflicts of interest.

## Data Availability

All data generated or analyzed during this study are included in this article.
